# Temporal changes in the spatial distribution of carabid beetles around arable field-woodlot boundaries

**DOI:** 10.1038/s41598-019-45378-7

**Published:** 2019-06-20

**Authors:** Michal Knapp, Miroslav Seidl, Jana Knappová, Martin Macek, Pavel Saska

**Affiliations:** 1 Department of Ecology, Faculty of Environmental Sciences, Czech University of Life Sciences Prague, CZ-165 00 Prague, Suchdol Czech Republic; 2 Institute of Botany, Czech Academy of Sciences, Zámek 1, CZ-252 43 Průhonice, Czech Republic; 3 Department of Botany, Faculty of Science, Charles University, Benátská 2, CZ-128 01 Prague 2, Czech Republic; 4 Crop Research Institute, Drnovská 507, CZ-161 06 Prague 6, Ruzyně Czech Republic

**Keywords:** Biodiversity, Agroecology

## Abstract

Carabids are considered beneficial arthropods in agroecosystems, where they prey on crop pests or consume weed seeds. Therefore, knowledge of the spatial distribution of carabids in agricultural landscapes is crucial to efficiently manage the ecosystem services that they provide. In the present study, we investigated the spatial distribution of carabids around arable field-woodlot boundaries in different seasons: (1) early spring, (2) late spring, (3) summer and (4) late autumn. The spatial distribution of carabid abundance (activity-density) and species richness varied seasonally, and the total abundance was highest within arable fields, except in early spring when it peaked at the boundaries. The observed pattern was mainly driven by the spatial distribution of the open-habitat species, which aggregated near the field boundaries during winter and early spring. The open-habitat species penetrated into woodlots during the summer season but occurred almost exclusively outside woodlots in the other sampling periods. The abundance of the forest species was highest within woodlots with the exception of the early spring season, when their abundance peaked at the boundaries. Carabid species richness was highest within arable fields in close proximity to woodlot boundaries with the exception of the summer season, when the total species richness was similar across habitats.

## Introduction

Human activities have significantly altered the environment at the global scale, and this process has been accelerated during the last century^1^. Many European landscapes have been exposed to agricultural intensification, with low-intensity farming systems being limited to small areas^2–4^. For example, agricultural landscapes cover approximately 53.5% of the total land area in the Czech Republic, and most is covered by intensively managed arable land (approx. 37.8% of the total area^5^). The originally diverse, highly mosaicked European landscapes have, in many cases, been converted into uniform areas consisting almost solely of intensively managed agricultural units, and these changes have resulted in the loss of biodiversity, at least at a local scale^2,6,7^.

The presence of non-crop habitats in intensively exploited agricultural landscapes is crucial for the survival of various animal taxa^7–11^, and many of them provide farmers with valuable ecosystem services, e.g., pollination^9^, pest^12^ or weed suppression^13^. Similar to many other animal taxa, carabid beetles inhabiting agricultural landscapes are strongly affected by landscape structure^10,14,15^, and they are considered beneficial organisms because they prey on crop pests or consume weed seeds^16,17^. Many species of carabids can be remarkably abundant in agroecosystems, so their contribution to ecosystem services is often noticeable^13,16^. However, to efficiently employ their services, knowledge of their spatial distribution in agricultural landscapes is of key importance.

Non-crop habitats within agricultural landscapes host species-rich and often abundant carabid assemblages that can spill over into adjacent arable fields and have the potential to suppress pest populations^12,16,18^. For example, temperate-forest specialist carabids may penetrate more deeply into arable fields than open-habitat specialists into woodlots^19^, and non-crop habitats are also temporarily utilized by carabids inhabiting neighbouring arable fields. The most obvious case is the aggregation of overwintering adults in the boundaries between arable land and non-crop habitats^20–22^. The majority of studies focusing on the spatial distribution of carabid assemblages in agricultural landscapes have not investigated the temporal variability in observed patterns, but those that did frequently revealed temporal variation in carabid spatial distributions^10,23–25^. The properties of arable fields dramatically change throughout the season due to various large-scale disturbances, e.g., tillage, harvest or pesticide application. Therefore, it is not surprising that seasonal changes in spatial distribution have been predicted for animals inhabiting such landscapes^26,27^. Interestingly, the existing data on seasonal changes in the distribution of carabid beetles around arable field-woodlot boundaries are limited.

In the present study, we investigated the spatial distribution of carabid beetles around field-woodland boundaries from early spring to autumn, and we were especially interested in the temporal variation in the relative distribution of carabid abundance and species richness near field-woodlot boundaries. Carabid species were classified according to their habitat specialization as open-habitat specialists, habitat generalists or forest specialists, and these groups were analysed separately to distinguish between the effects attributable to group. We hypothesized that all groups (open-habitat specialists, habitat generalists and forest specialists) accumulate at the field-woodlot boundaries during early spring and late autumn because habitat edges are suitable overwintering sites for ground beetles^20–22^. We assumed weak penetration of forest species into arable fields because edges subject to anthropogenic interventions are impenetrable for forest species, thus preventing their dispersal^28^. Finally, we hypothesized that open-habitat specialists spill over into adjacent woodlots during summer and autumn as a result of agricultural operations (disturbances) in arable land^27^.

## Results

In total, 10 270 carabid beetles of 83 species were collected during the study, and our analyses were based on the final dataset of 8396 carabid beetles of 76 species from the four sampling periods with completely undamaged samples (for details, see Tables S1 and S2 in the Supplementary Material).

### Abundance (activity-density)

**Figure 1:**
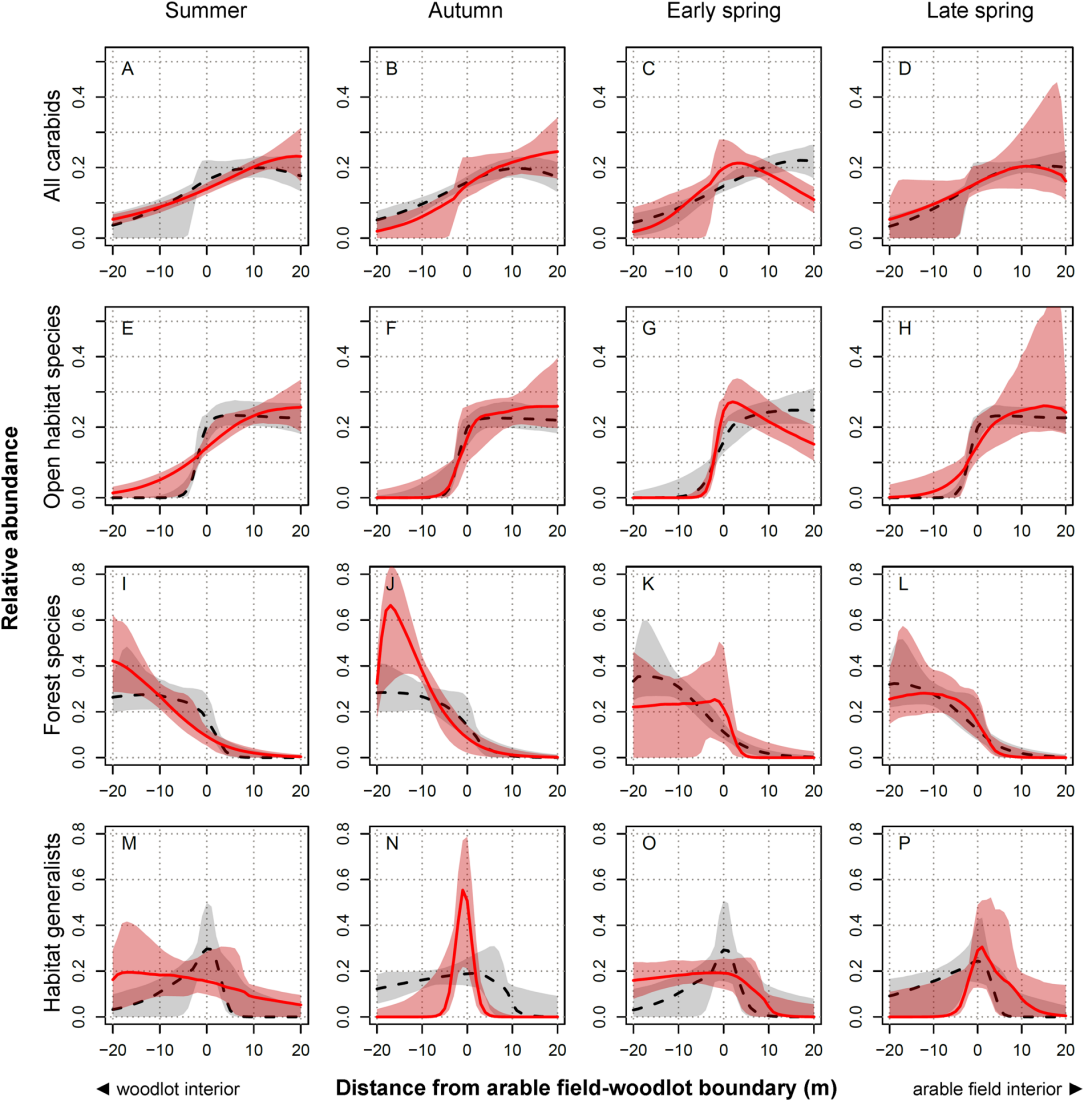
Temporal changes in the relative distribution of carabid beetle abundance around arable field-woodlot boundaries. Data are shown for all carabid beetles combined (panel A–D), open-habitat species (panel E–H), forest species (panel I–L) or habitat generalists (panel M–P) and for each of four sampling periods: summer season (**A**,**E**,**I**,**M**), autumn season (**B**,**F**,**J**,**N**), early spring season (**C**,**G**,**K**,**O**) and late spring season (**D**,**H**,**L**,**P**). The red line indicates the median relative distribution of abundance in a given period, and the corresponding 95% confidence interval is shown as the pink area. The black dashed line indicates the median relative distribution of abundance pooled across all other periods, and the corresponding 95% confidence interval is shown as the grey area. A significant difference in the relative distribution of carabid abundance in a given period compared to the median distribution across the other three periods is indicated by a lack of overlap between the red line and the grey area within a panel.

In general, the total carabid abundance increased from the woodlot interior towards the arable field, but significant temporal variation was observed (Fig. 1A–D). Total abundance shifted more to the field interior in summer and autumn (July and November sampling periods; Fig. 1A,B), whereas the total abundance peaked around the field-woodlot boundary in early spring (April sampling period; Fig. 1C). The overall spatial distribution pattern observed for the total carabid abundance was mainly driven by the spatial distribution of the open-habitat species (compare panels A–D with E–H in Fig. 1). The open-habitat species dominated our dataset, and the numbers of carabid specimens collected in arable fields were significantly higher than those in woodlots (for details, see Fig. S2 in the Supplementary Material). The abundance of the open-habitat species sharply increased at the ecotone (field-woodlot boundary) throughout the whole year, except in the summer (July) when the open-habitat species also penetrated more deeply into the woodlot interior and the increase in their abundance towards the arable field interior was more linear (Fig. 1E). The abundance of the open-habitat species was highest within arable fields with the exception of early spring, when their abundance peaked around the field-woodlot boundary. The spatial distribution of the abundance of the forest species also fluctuated (Fig. 1I–L); these species moved deeper into the woodlot interior in summer and autumn (Fig. 1I,J) and reached the field-woodland boundary in spring (Fig. 1K,L). The abundance of the habitat generalists peaked around the field-woodlot boundary, mainly in autumn and late spring (Fig. 1N,P), but the observed patterns could be affected by the limited data available for this group (the habitat generalists represented a small portion of the individuals collected in this study; see Table S1 in the Supplementary Material).

### Species richness

**Figure 2:**
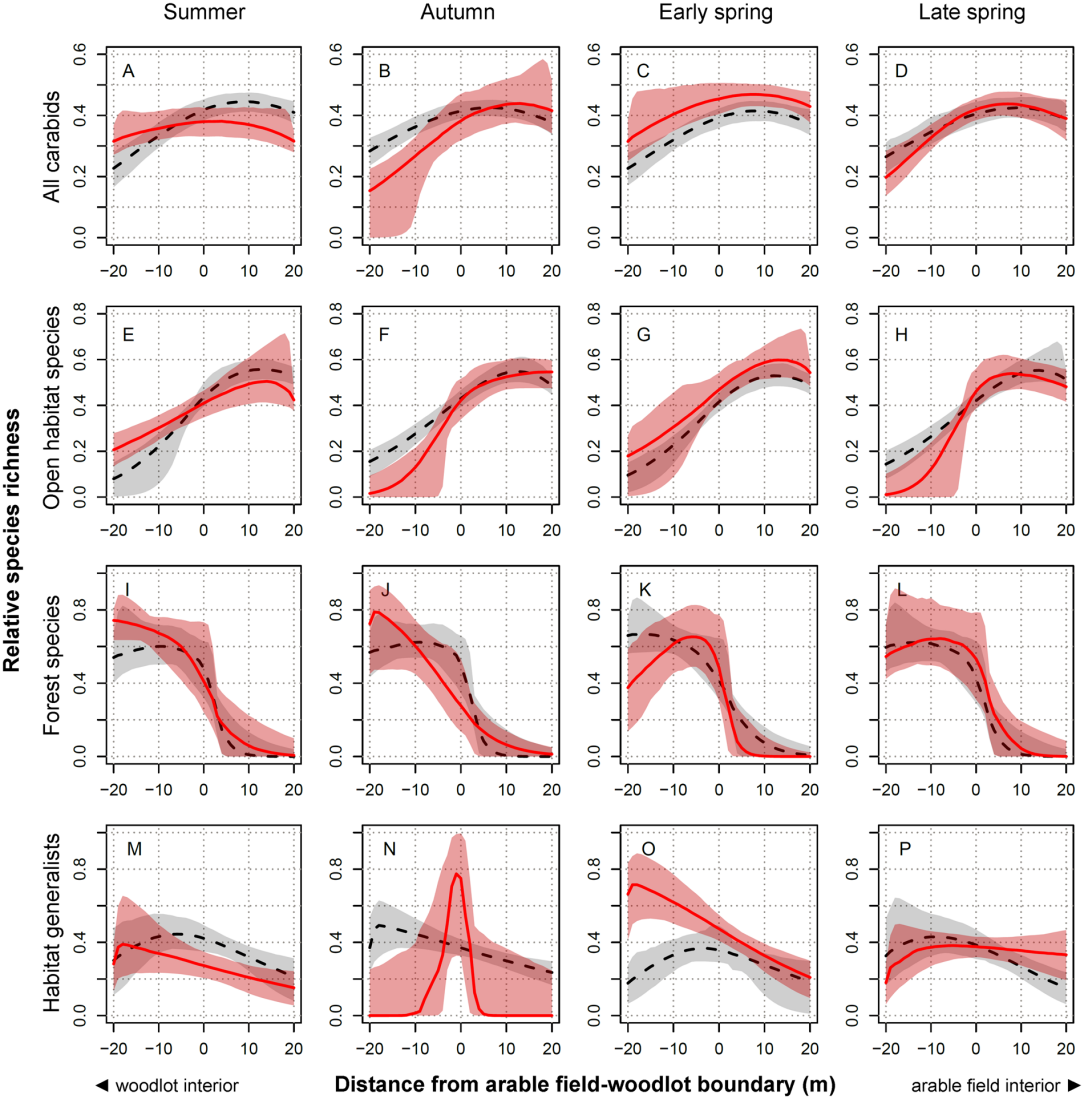
Temporal changes in the relative distribution of carabid beetle species richness around arable field-woodlot boundaries. Data are shown for all carabid beetles combined (panel A–D), open-habitat species (panel E–H), forest species (panel I–L) or habitat generalists (panel M–P) and for each of four sampling periods: summer season (**A**,**E**,**I**,**M**), autumn season (**B**,**F**,**J**,**N**), early spring season (**C**,**G**,**K**,**O**) and late spring season (**D**,**H**,**L**,**P**). The red line indicates the median relative distribution of species richness in a given period, and the corresponding 95% confidence interval is shown as the pink area. The black dashed line indicates the median relative distribution of species richness pooled across all the other periods, and the corresponding 95% confidence interval is shown as the grey area.

In general, total carabid species richness slightly increased from the woodlot interior towards the field-woodlot boundary (Fig. 2A–D), but significant temporal variation was apparent in this pattern. Carabid species richness was almost even along the investigated field-woodlot gradient in summer (Fig. 2A), whereas significantly more carabid species were collected in arable fields than in woodlots in autumn and late spring (Fig. 2B,D). Similar to abundance, the spatiotemporal patterns of total species richness were mainly driven by open-habitat species, which was very low in the woodlot interior and increased sharply at the field-woodlot boundary in autumn and late spring (Fig. 2F,H). In contrast, relatively higher proportions of open-habitat species were recorded in woodlots, and only moderate increases in open-habitat species richness towards arable fields were observed during summer and early spring (Fig. 2E,G). The species richness of the forest species was highest deep in the woodlot interior in summer and autumn (Fig. 2I,J), but it peaked near the field-woodlot boundary in early spring (Fig. 2K). Various patterns in the spatial distribution of generalist species richness were observed across the seasons (Fig. 2M–P), but this could have been partly due to the limited presence of generalist species in our dataset.

### Species composition

**Figure 3:**
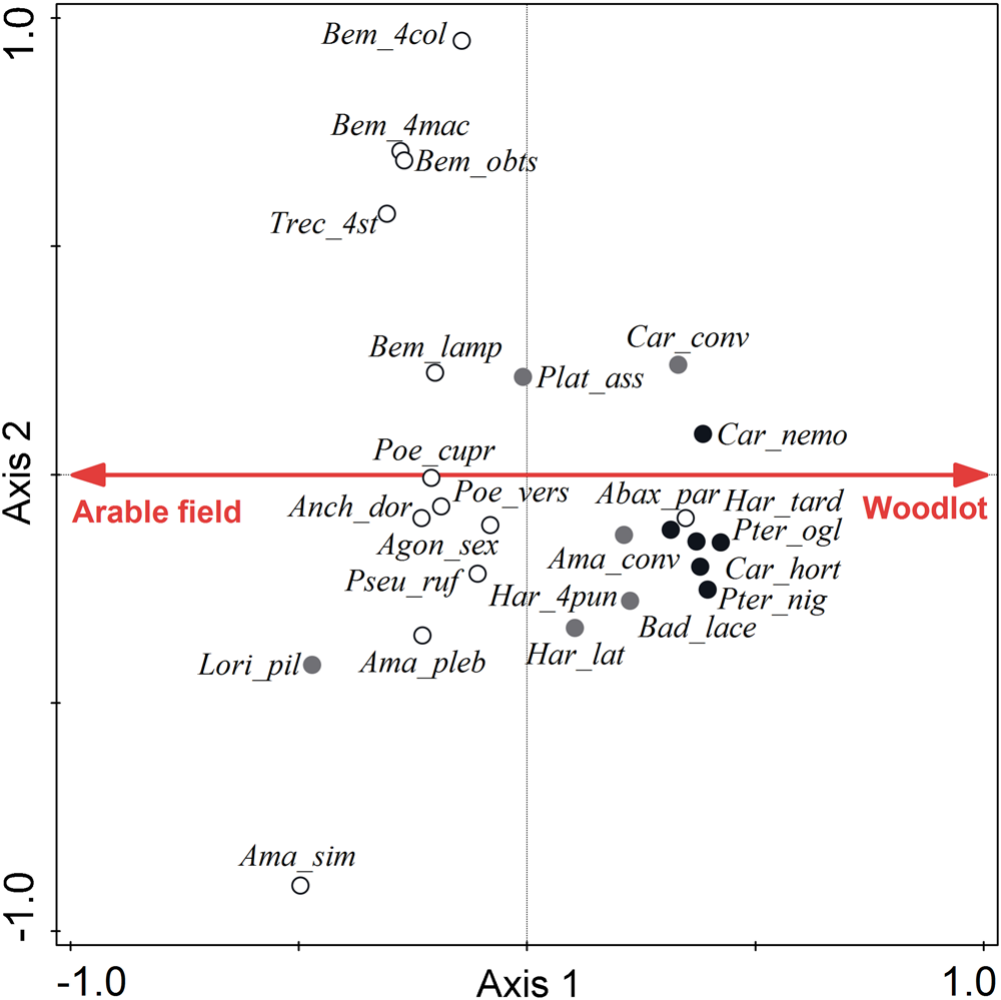
Spatial distribution of carabid species around arable field-woodlot boundaries. The ordination diagram shows a general spatial distribution of carabid species across all seasons (data for summer, autumn, early spring and late spring seasons are merged). The first axis represents the gradient between an arable field interior (20 m from the habitat boundary) and a woodlot interior (20 m from the habitat boundary). White circles represent open-habitat specialist species; grey circles represent generalist species; and black circles represent forest specialist species. The classification is based on data published in the book by Hůrka^45^. The first axis explained 9.2% of the total variability in the species data (note that this value represents the net spatial variation along the field-woodlot gradient corrected for the effects of transect identity and the temporal variation among seasons). Species names are abbreviated; for full species names, see Table S1 in the Supplementary Materials.

**Figure 4:**
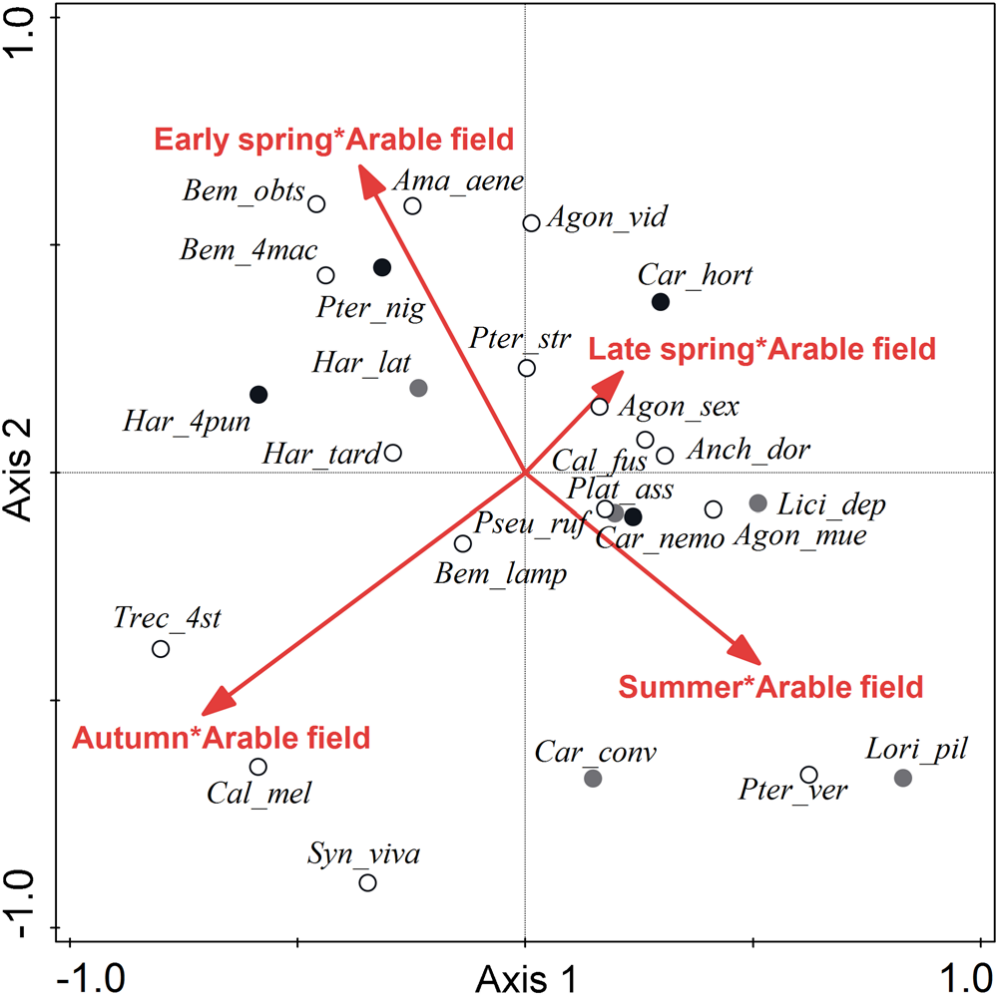
Temporal changes in the spatial distribution of carabid assemblages around arable field-woodlot boundaries. The position of the species relative to the central part of the ordination diagram indicates temporal changes in their spatial occurrence. The position of a given species in the direction of a particular red arrow denotes a shift in the species optimum towards the arable field interior in a given sampling period; e.g., the optimum of *Carabus hortensis* (*Car_hort*) was shifted more towards the arable field (but still within the woodlot) in late spring. White circles represent open-habitat specialist species; grey circles represent generalist species; and black circles represent forest specialist species. The classification is based on data published in the book by Hůrka^45^. The first axis explained 3.0% and the second axis 2.4% of the total variability in the species data (note that this value represents the net effects of the interaction between space and time on the species composition of carabid assemblages corrected for the effects of transect identity, time and space). Species names are abbreviated; for full species names, see Table S1 in the Supplementary Materials.

The species composition of carabid assemblages varied significantly along the field-woodlot gradient (partial CCA: pseudo-F = 10.8, P = 0.002, net variance explained = 9.2%). Different species dominated the assemblages collected within arable fields and within woodlots, and the occurrence of particular carabid species corresponded well to the published information on their habitat preferences (Fig. 3). Interestingly, the optima for some open-habitat specialist species occurred near the field-woodlot boundary rather than within the arable field, whereas the optima of the generalist species was also quite near the field-woodlot boundary rather than evenly scattered along the arable field-woodlot gradient. The spatial variability in the species composition of the carabid assemblages was unstable in time (significant interaction between sampling time and the spatial variability of the carabid assemblages; partial CCA: pseudo-F = 2.7, P = 0.002, net variance explained = 7.4%), indicating that species optima along an arable field-woodlot gradient shifted during the season; e.g., the occurrence of *Trechus quadristriatus* shifted more towards the arable field interior during autumn (Fig. 4). The degree of temporal variation in the species composition of carabid assemblages differed between those originating from various positions along the arable field-woodlot gradient; i.e., the effect of the distance × period interaction was significant (db-RDA: pseudo-F = 3.0, P = 0.002, net variance explained = 8.2%). In particular, assemblages originating from the woodlot interior (10 or 20 m from the habitat boundary) followed different trajectories than assemblages originating from arable land and the habitat boundary, i.e., the ecotone (see Fig. S3 in the Supplementary Material). This corresponds well to the observed higher temporal variability in the occurrence of the open-habitat species and the habitat generalists compared to the forest species (see Fig. S4 in the Supplementary Material).

## Discussion

Our results clearly showed that the spatial distribution of carabid beetles in agricultural landscape varied over time. This spatiotemporal variation was mainly driven by open-habitat species, whereas the distribution of forest species was more stable in time. The most striking difference in the spatial distribution of open-habitat species occurred between the early spring period and the following growing season, which is supposedly a result of carabid overwintering behaviour. Interestingly, some generalist species behaved as ecotone specialists in our study system.

The total abundance of carabid beetles was higher within arable fields than within neighbouring woodlots, and this difference between arable fields and non-crop habitats was also observed in a previous study in the same area^10^ as well as in other areas^29^. The productivity of arable fields in temperate regions is maximized by extensive fertilization and is typically higher than in non-crop habitats^30^, and carabid abundance seems to be related to habitat productivity^29^. The dominance of open-habitat species in the landscape is a possible cause of their leading role in determining the spatiotemporal dynamics of carabid assemblages in our study system; they spilled over into woodlots during the summer season and drove the accumulation of carabid abundance at the ecotone in early spring. It is plausible that the spillover from arable fields to woodlots will be even more apparent during the late summer season, i.e., after crop harvest. The effects of such dramatic changes in habitat properties on the spillover of various animals have been hypothesized as a general pattern by Rand *et al*.^27^ and have also been directly shown for carabids by Schneider *et al*.^31^. Unfortunately, our samples from August were partially destroyed by flooding and an accident, so we could not test this.

The most significant spatial redistribution of carabid beetles in agricultural landscapes takes place before the winter. Many carabid species that breed within arable land overwinter as adults in neighbouring non-crop habitats^20,21^. When overwintering, open-habitat carabids accumulate at habitat edges and only rarely penetrate deeply into non-crop habitats^22^, resulting in incredibly high winter population densities (up to 200 individuals per m^2^)^21,22^. After overwintering, open-habitat carabids colonize arable field interiors^25^. Our data on the spatial distribution of open-habitat species during the early spring season corresponded well to this pattern. Interestingly, the abundance of forest species at woodlot-arable field boundaries in our study area was significantly higher during the early spring season than during other sampling periods; it seems that even forest species may aggregate near forest edges when overwintering^22^. Carabid beetle aggregation near habitat boundaries during winter season, was also observed at grassland-forest boundaries by Ohwaki *et al*.^32^.

We did not observe substantial spillover of forest species into neighbouring arable fields during any of the sampling periods, and such a process has been proposed as a mechanism responsible for the positive effects of non-crop habitat proximity on the diversity and abundance of predatory arthropod species within arable fields that results in enhanced pest regulation^33^. Consistent with our results, Ferrante *et al*.^34^ measured rates of predation on artificial caterpillars around field-woodlot boundaries in Argentina and did not observe enhanced predation by chewing insects in proximity to woodlots, but the majority of other studies have provided contrasting evidence, observing deeper or similar levels of penetration by forest species into arable fields as that of open-habitat species into forests^19,35^. The differences between the results of particular studies could be related to contrasts in habitat productivity, which positively relates to the abundance of food sources for carabids. Soils in woodlots within our study area were shallow and poor in nutrients and thus contrasted with the high productivity of the surrounding arable fields. The contrast was probably much lower in the study system investigated by Roume *et al*.^19^, as the carabid activity-densities recorded within those arable fields and woodlots were comparable. An alternative explanation can be differences in agricultural practices between particular study systems, e.g., the application of higher amounts of pesticides can result in lower carabid abundances within arable fields. Carabid edge responses also seem to be driven by habitat edge properties: forest species tend to avoid edges with anthropogenic, highly disturbed open habitats, so their penetration into arable fields is less common than that into natural open habitats, e.g., various grasslands^28^.

Accumulating evidence of the importance of edge effects for carabids, including the results of our study, indicates that carabid spillover into adjacent habitats only acts over very short distances. Open-habitat species commonly penetrate only a few metres into forest habitats, whereas forest species rarely disperse more than a few tens of metres into open habitats^19,32,35–41^. This is likely to reflect the relatively strict habitat preferences of many carabid species^42^, and there is evidence that environmental filtering contributes to the formation of carabid assemblages^43,44^. Moreover, generalist carabid species are commonly not abundant in agricultural landscapes^16,22,45^. As proposed by Rand *et al*.^27^, massive spillover between crop land and neighbouring non-crop habitats is only possible if focal species are able to prosper/survive in both habitat types. Therefore, the strict habitat requirements and limited dispersal abilities of many carabid species^42,45^ are a probable reason for the very short-range edge effects observed. More mobile groups of beneficial insects, e.g., pollinators, seem to respond more strongly to the presence of non-crop habitats in agricultural landscapes^46^.

As agricultural landscapes are highly dynamic systems, mainly due to large-scale disturbances in arable fields, high spatiotemporal variability in insect distributions can be expected^27^. Interestingly, the majority of studies investigating the spatial distribution of carabid beetles in agricultural landscapes have not considered the temporal variation. Data from various sampling periods are routinely pooled for statistical analyses^19,36,39^, or the temporal variation in the spatial distribution is only investigated for part of the season, like summer^23^. Studies investigating carabid responses to forest edges have predominantly focused on forest-grassland boundaries, while forest-arable land boundaries have been poorly investigated (see the low number of such studies in the meta-analysis Magura *et al*.^28^. Therefore, studies investigating the spatiotemporal variation in the distribution of carabids around arable field-woodlot boundaries are greatly needed as they may provide useful information for the practical management of carabid populations and thus the ecosystem services that they provide. According to our results, field-woodlot ecotones should remain undisturbed from late autumn to early spring as the majority of adult carabids aggregate there.

## Methods

### Sampling site

Carabid beetles (Coleoptera: Carabidae) were collected from two large arable fields located 6 km south of the town of Sedlčany between the villages of Nedrahovické Podhájí, Rovina and Vysoký Chlumec in the Czech Republic (the GPS coordinates of the centre of each investigated field are: N 49°36.497′, E 14°24.321′ and N 49°36.499′, E 14°24.939′, respectively). These fields are exceptional due to the numerous woodlots of various sizes within the arable land, some of which are greater than 50 m in diameter (see Figure S1 in the Supplementary Material for an aerial photograph of the study area). The terrain of the woodlots was slightly than the arable land and was covered by shrubs and trees. The most abundant shrub species were black elder (*Sambucus nigra*), snowberry (*Symphoricarpos albus*) and wild roses (*Rosa* spp.), and the most numerous trees were oaks (*Quercus robur* and *Quercus petraea*), black locust (*Robinia pseudoacacia*), Scots pine (*Pinus sylvestris*) and ash (*Fraxinus excelsior*). The woodlots also included large stones that probably precluded cultivation of these areas in the past. The two investigated arable fields were planted with different crops during the sampling periods; winter barley (*Hordeum vulgare*) and winter wheat (*Triticum aestivum*) were planted in 2013, and winter barley and winter rape (*Brassica napus*) in 2014. Crop identity is known to affect carabid beetle abundance^47^ and thus relative distribution of abundance within fields rather than absolute values were analysed. Both fields were conventionally managed, and pesticides and fertilizers were applied at frequencies and doses common for particular crops in the Czech Republic (yearly, an average of 400 kg of N and 100 kg of P_2_O_5_ applied per ha and approx. 8 pesticide applications).

### Data collection

**Figure 5:**
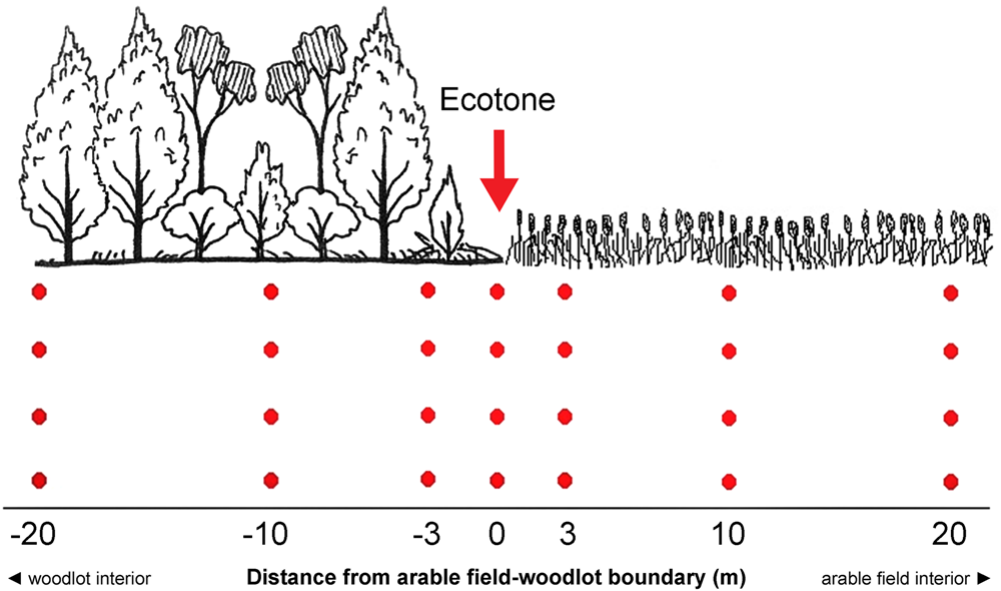
Sampling design. Spatial distribution of pitfall traps (represented by red dots) along a transect perpendicular to an arable field-woodlot boundary. Within each distance from the field-woodlot boundary, four pitfall traps were deployed with a spacing of three metres. Four independent transects were replicated in the study area.

To investigate the spatiotemporal variation in the abundance (number of collected individuals; as this value is affected not only by population density but also by animal movement activity term “activity-density” is employed in some studies), species richness and species composition of carabid assemblages around field-woodlot boundaries, carabid beetles were sampled using pitfall traps. Pitfall traps were arranged along four transects (two per investigated field) that crossed the boundary between the arable land and the neighbouring woodlot. We selected the largest available to ensure that the transects within the woodlots did not approach the opposite edges. Each particular transect consisted of 7 sampling sites: at the edge and at distances of 3, 10 and 20 m towards the interiors of both the fields and woodlots. In each sampling site, we used 4 traps arranged in a row with a spacing of 3 m between the traps (Fig. 5). In total, 112 pitfall traps were operated simultaneously during each sampling period.

The pitfall traps were made of 0.4-litre plastic cups (10 cm in diameter) buried in the soil with the rim of the cup flush with the soil surface. The cups were filled with 150 ml of preservative fluid made from a propylene glycol water solution (dilution 1:3), which seems to be efficient for collecting carabid beetles and is relatively non-toxic to non-target organisms^48^. Traps were covered by roofs made of an aluminium sheet to prevent flooding during heavy rainfall and to protect the traps from damage by large mammals (e.g., wild boars and roe deer). Roofs were placed 5 centimetres above the openings of the cups using three long nails that penetrated the corners of the roofs.

Carabid beetle surveys covered the entire season; five discrete sampling campaigns were repeated in 2013 and 2014 to provide a complex picture of the seasonal variation. The timing of particular sampling periods was selected to match the major phenological phases of crop development (crop growth, crop ripeness, harvested field, tilled field, newly emerged overwintering seedlings) and to avoid conflicts with agricultural management operations. The exact sampling dates were as follows: 1^st^ sampling period (“summer”) = 18^th^ June to 9^th^ July 2013; 2^nd^ sampling period (“late summer”) = 24^th^ July to 5^th^ August 2013; 3^rd^ sampling period (“autumn”) = 15^th^ October to 8^th^ November 2013; 4^th^ sampling period (“early spring”) = 14^th^ March to 18^th^ April 2014; and 5^th^ sampling period (“late spring”) = 19^th^ April to 27^th^ May 2014.

### Pitfall trap sample processing

After removing surplus preservation fluid by sieving, the samples from each pitfall trap were placed into plastic bags, coded and transferred to the laboratory at the Czech University of Life Sciences in Prague. Samples were subsequently stored in a freezer until processing. Later, all carabid beetles were sorted from the pitfall trap samples and identified to species based on morphological characters using the identification guide by Hůrka^45^.

### Statistical analyses

#### Final dataset

For the purpose of the statistical analyses, samples from 4 pitfall traps within a particular sampling site were combined, which resulted in 28 pooled samples per sampling period. This was done because individual traps could have been affected by fine-scale microhabitat conditions in their close surroundings, and stochastic processes could have strongly affected the species composition of smaller samples. Moreover, particular traps within a sampling site were close to each other and could be considered pseudo-replicates. Therefore, the pooled samples provided a more complex picture of the assemblages inhabiting the given distances from the habitat boundary. As our sampling distances are also close to each other, there is a spatial autocorrelation present. To cope with this fact, adequate analytic tools were employed (the Huismann-Olff-Fresco models; see below for details). It is important to note that several traps were destroyed in the 2^nd^ sampling period (late summer) by flooding and by a tractor unexpectedly crossing the field. We excluded data from this sampling period from the final dataset and performed statistical analyses with a limited dataset consisting of completely undamaged samples. The final dataset included the following four sampling periods: summer (4079 individuals/60 carabid species collected), autumn (650 individuals/33 species), early spring (1638 individuals/53 species) and late spring (2029 individuals/56 species); for details, see Table S1 in the Supplementary Material. For the subsequent analyses, carabid species were classified according to their habitat preferences as open-habitat species, forest species and habitat generalists based on published information^45^.

#### Relative abundance and species richness

In the analyses, we focused on seasonal changes in the spatial distribution of relative rather than absolute carabid abundance and species richness because the numbers of collected carabid individuals and species per sample were incomparable across the four sampling periods as the duration of the sampling periods varied (from 21 to 38 days). Additionally, the movement activity of carabid beetles varies strongly with temperature, so daily pitfall trap captures during early spring and late autumn are much lower than those during the summer season^49^.

The relative abundance for a given sample was calculated as the number of individuals in the sample divided by the total number of individuals collected in all samples for a given transect and sampling period. Therefore, the relative abundance values ranged from 0 to 1 (a value of 0 meant that none of the individuals collected along a given transect in a given period originated from the focal sample, and a value of 1 indicated that all individuals collected along a given transect in a given period originated from the focal sample). Relative abundance was calculated for the entire community (all carabids) as well as separately for open-habitat species, habitat generalists and forest species. Relative species richness was calculated in the same way as relative abundance but using species counts instead of specimen counts. It is important to note that, in contrast to the presence of a specimen, the presence of a given species in a sample is not exclusive; i.e., the same species can be present at more sampling distances along a given transect in a given sampling period. Thus, the sum of all relative species richness values across a given transect for a given period can be higher than 1 (the maximal theoretically possible value is 7, but the real values in our study were close to 1).

The temporal variation in the spatial distribution of the relative carabid beetle abundance and species richness along the transects was explored using logistic curves with pre-defined fundamental, ecologically meaningful shapes: the Huismann-Olff-Fresco models (HOFs)^50^ as implemented in the eHOF package in R^51,52^. HOFs are frequently employed to investigate species responses along environmental gradients especially in plant ecology^51,53^. Here, we used geographical distance from the arable field-woodlot boundary as the environmental gradient, and we constrained the evaluated model types to five basic response shapes: flat (type I), linear (type II), plateau (type III), unimodal symmetric (type IV) and asymmetric (type V). To explore the temporal changes in the spatial distribution of relative abundance and relative species richness along a gradient from woodlot interior to field interior, we fitted separate HOFs for each sampling period (summer, autumn, early spring, and late spring). To check if the spatial distribution during a particular period differed from that during other periods (pooled data for the other three periods), we obtained a median response curve (one for the focal period and one for the other periods) and 95% confidence intervals by bootstrapping. We fitted HOF curves based on 499 randomized sets of values sampled using a permutation scheme with replication values from different transects while keeping the number of observations for each distance from the field-woodlot boundary constant. For each bootstrapped sample, we selected a type of HOF model that minimized model deviance, and we then calculated the median responses and 95% confidence intervals of the bootstrapped values and explored the results graphically. Analyses were performed separately for both response variables (relative activity density and relative species richness) and for various species groups: (1) all carabid species, (2) open-habitat species, (3) habitat generalists, and (4) forest species. Input data for these analyses and the R script are available in Table S2 and R script file in the Supplementary Material, respectively.

#### Species composition

To analyse the spatiotemporal variation in the species composition of carabid assemblages around field-woodlot boundaries, multivariate ordination techniques were applied. Prior to analyses, species data were log-transformed (log_10_(x + 1)). Rare species represented by less than 4 collected specimens were excluded from the analyses, resulting in a final dataset of 55 carabid species. Unimodal analyses were appropriate for our data as they automatically include data standardization; i.e., changes in relative species composition were analysed, not changes in sample sizes^54^.

To analyse general spatial patterns in the species composition of carabid assemblages along a distance gradient perpendicular to the field-woodlot boundary, canonical correspondence analysis (CCA) was employed, and the significance of the distance to the field-woodlot boundary was tested using a permutation test. The distance to the field-woodlot boundary was used as an independent environmental variable; transect identity was used as a class-type covariate; and a permutation scheme was established to only allow samples to be permutated within a particular transect within a particular sampling period. This restricted permutation scheme resulted in 16 blocks (unique transect × sampling period combinations), between which samples were not exchanged during the permutation test.

To analyse temporal changes in the spatial distribution of the species composition of carabid assemblages, i.e., the interaction between sampling time and the distance to the field-woodlot boundary, CCA was employed, and the significance of the interaction was tested using a randomization test. The interaction between time and distance to the field-woodlot boundary was used as an independent environmental variable, and transect identity, time and distance to the field-woodlot boundary were used as covariates. A permutation scheme was established to allow samples to be permutated within a particular transect as well as to permute the identity of the sampling period. This restricted permutation scheme thus resulted in 16 blocks (unique transect × sampling period combinations), within which samples were exchanged during the permutation test, and sampling period identities were exchanged within a particular transect.

To compare the temporal variability in assemblages sampled at different distances from the arable field-woodlot boundary, distance-based redundancy analysis (db-RDA) was employed. In the first step, principal coordinate analysis (PCoA) was performed, and scores were calculated based on Bray-Curtis distances^55^. These scores express the dissimilarity of sampled assemblages. PCoA scores were then used as response variables; distance and sampling period were used as covariates; and the distance × period interaction was used as an explanatory variable. For the permutation test, the transect was used as a block.

Permutation tests, which were applied to direct multivariate analyses, i.e., CCAs and db-RDA, were performed using the original data matrix (species × environment) plus 499 permutations. Data which served as inputs for these analyses are available as Appendix C in the Supplementary Material. All multivariate analyses were performed using Canoco for Windows 5.0 software^54^.

## Supplementary information

Supplementary Figures S1-S4 + R Script

Table S1

Table S2

## Data Availability

Analysed data are enclosed in the manuscript as Supplementary Material File.
