# Supplementary material for: Temporal changes in the spatial distribution of carabid beetles around arable field-woodlot boundaries
Source: Sci Rep. 2019 Jun 20;9:8967. doi: 10.1038/s41598-019-45378-7 (PMC6586664; doi:10.1038/s41598-019-45378-7)
Supplement: Supplementary file 1 — Supplementary Figures S1-S4 + R Script [file 41598_2019_45378_MOESM1_ESM.pdf]

Scientific Reports

Temporal changes in the spatial distribution of carabid beetles around arable field-woodlot boundaries

Michal Knapp[1@], Miroslav Seidl[1], Jana Knappová[2,3], Martin Macek[2,3], Pavel Saska[1,4]

[1] Department of Ecology, Faculty of Environmental Sciences, Czech University of Life Sciences Prague, CZ-165 00, Prague - Suchdol, Czech Republic; knapp@fzp.czu.cz, seidlm@fzp.czu.cz

[2] Institute of Botany, Czech Academy of Sciences, Zámek 1, CZ-252 43, Průhonice, Czech Republic; jana.knappova@ibot.cas.cz, martin.macek@ibot.cas.cz

[3] Department of Botany, Faculty of Science, Charles University, Benátská 2, CZ-128 01, Prague 2, Czech Republic

[4] Crop Research Institute, Drnovská 507, CZ-161 06, Prague 6 - Ruzyně, Czech Republic; saska@vurv.cz

Supplementary material:

Figures S1 – S4

R Script

Figure S1 - Aerial photograph of the study area. This figure is not covered by the CC BY licence. [Source: www.mapy.cz< http://www.mapy.cz >]. All rights reserved, used with permission. Drawings were added in Photoshop 5.0 software.

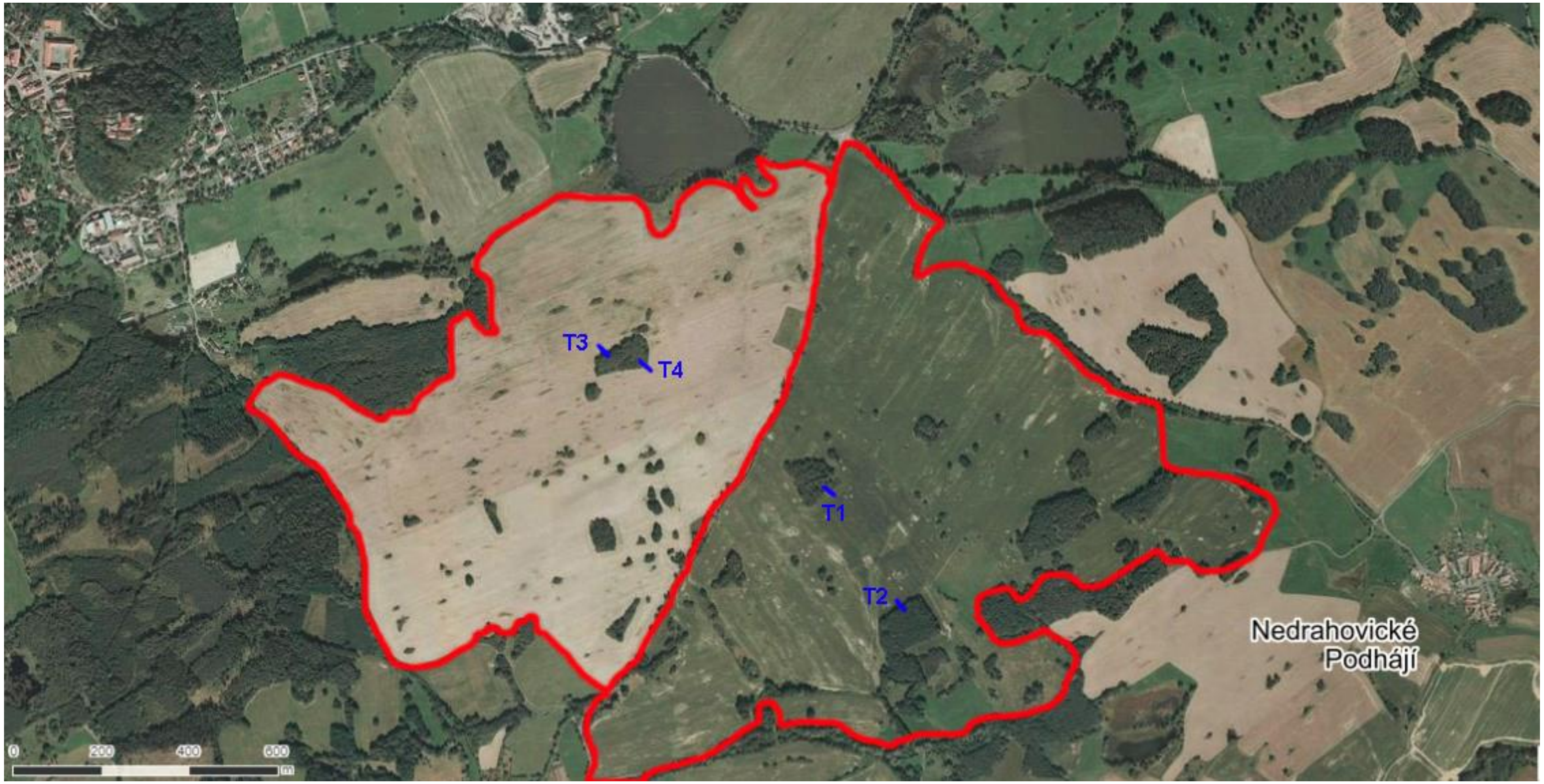

Knapp et al. – Scientific Reports

Figure S2 - Comparison of carabid abundance (activity-density) between woodlot and arable field interiors

```
> m<-glmmPQL(N~Period*Position, random =~ 1|Transect, family=quasipoisson, data=dat)

> anova.lme(m, test="F")
               numDF denDF  F-value p-value
(Intercept)        1    21 621.4771  <.0001
Period             3    21  10.1275  0.0002
Position           1    21  16.0571  0.0006
Period:Position    3    21   0.3696  0.7757
```

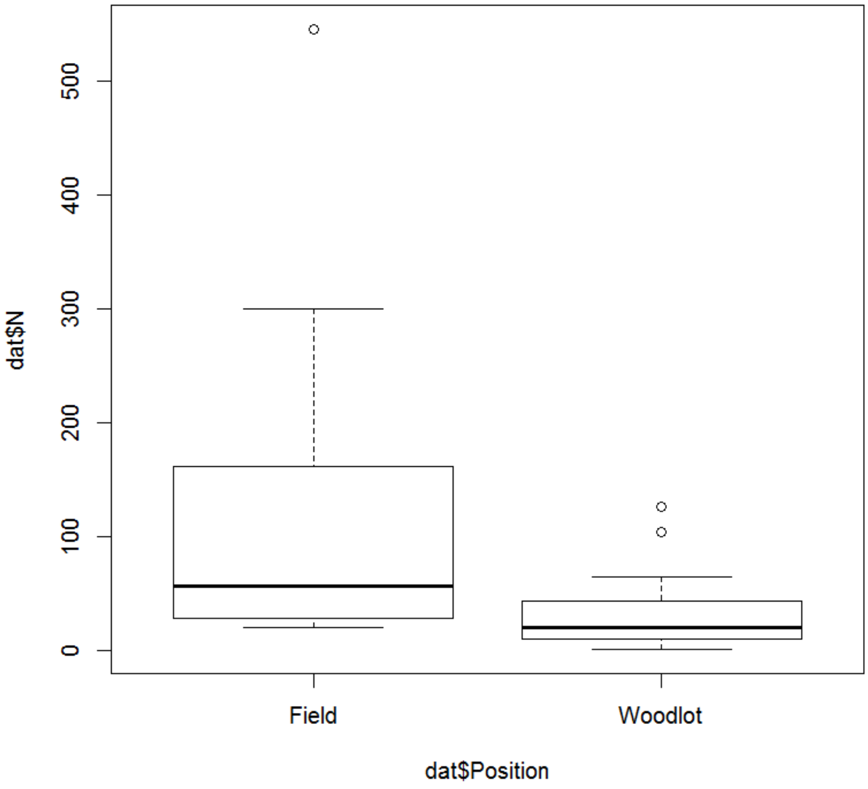

Figure S3 - Temporal shifts in species composition of carabid assemblages originating from various sampling sites (positions on transects)

For the purpose of visualisation of temporal shifts in carabid assemblages originating from particular distances, unconstrained unimodal analysis DCA was performed. Samples were classified according to the position on a transect (-20 m = yellow, -10 m = orange, -3 m = red, 0 m = brown, 3 m = light green, 10 m = medium green, 20 m = dark green) within resulting ordination diagram and these originating from the same transect were connected by lines to highlight temporal changes of respective carabid assemblages.

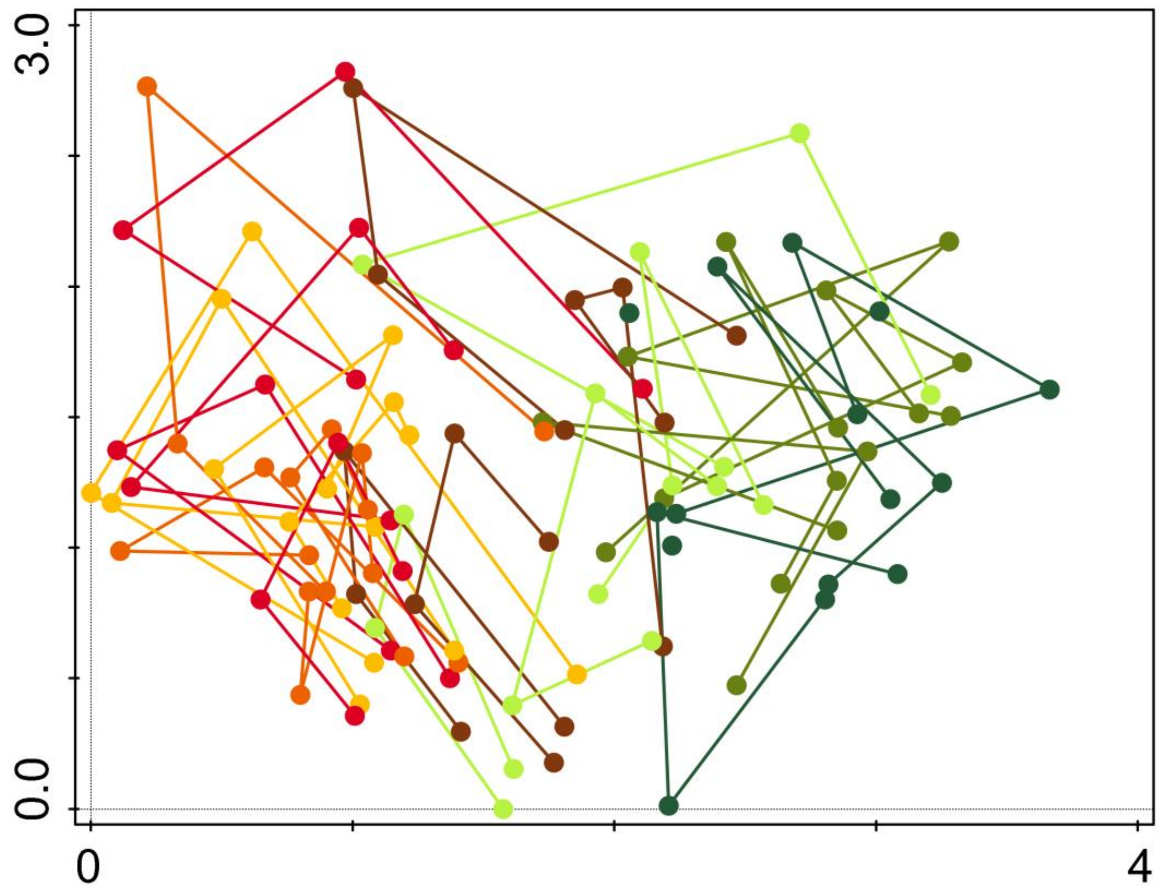

Figure S4 - Net effect of time on species composition of carabid assemblages

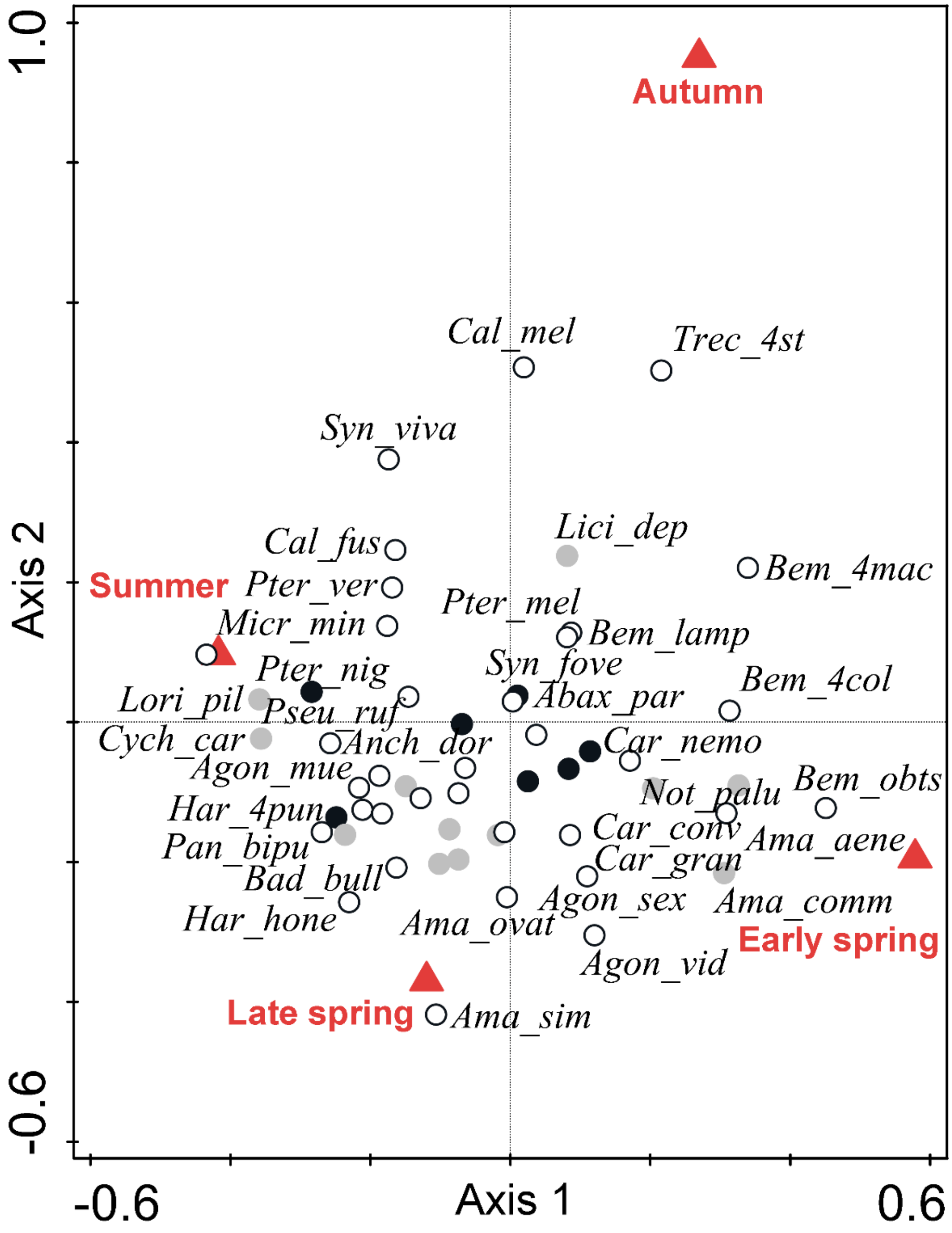

Knapp et al. – Scientific Reports

R script

```
### Supplementary material to the manuscript#
### Temporal changes in the spatial distribution of carabid beetles
around arable field-woodlot boundaries --------
### code written by Martin Macek martin.macek@ibot.cas.cz

#code written and used under R 3.2.5 version
library(eHOF) #using eHOF version 1.7

carabid <- read.table("carabid.csv",sep=";",head=T) #read data from file
str(carabid)  #check table structure


#carabid activity
#compares spatial structure of activity of carabid beetles (using
relative numbers - e.g. actual number of individuals at given sample
divided by transect total at given sampling period)

month.sel <- c(7,10,4,5) # select sampling periods for pairwise
comparison
bootnr <- 499 #set number of permutations
x <- unique(head.sp$Distance)
set.seed(324)
types <- c("I","II","III","IV","V")

#relative activity
variables <- c("std.count","o.count","f.count","g.count") #select
variable of interest: relative activity
var.names <- c("All carabids","Open habitat species","Forest
species","Habitat generalists")

#relative diversity
variables <- c("total.prop","open.prop","for.prop","gen.prop") #select
variable of interest: relative diversity
var.names <- c("All carabids","Open habitat species","Forest
species","Habitat generalists")

#prepare multiple-plot window
widths <- rep(1,length(variables))
widths[1] <- 1.1 # make first column 10% wider
heights <- rep(1,length(month.sel))
heights[length(month.sel)] <- 1.1 # make last row 10% wider


#pdf("relative_activity.pdf",8,8) #inicialize pdf device (relative
activity)
pdf("relative_diversity.pdf",8,8)  #inicialize pdf device (relative
diversity)

layout(mat=matrix(data =
1:(length(variables)*length(month.sel)),nrow=length(variables),ncol=4,byr
ow=T),widths=widths,heights=heights)

for(j in 1:length(variables)){
  variable <- variables[j]
```

```
  set.seed(324)
    for(k in 1:4){
      pair <- month.sel[-k] #rest of the year to be compared with
selected month
      hof.pred <- matrix(data= NA, nrow = length(-20:20), ncol = bootnr)
#create matrix to store bootstrapped values
      for(i in 1:bootnr){subs <- c(sample(which(carabid$Distance == x[1]
& carabid$Month %in% pair),12,replace=T),   #sample values with
replacement for each transect position
                                   sample(which(carabid$Distance == x[2]
& carabid$Month %in% pair),12,replace=T),
                                   sample(which(carabid$Distance == x[3]
& carabid$Month %in% pair),12,replace=T),
                                   sample(which(carabid$Distance == x[4]
& carabid$Month %in% pair),12,replace=T),
                                   sample(which(carabid$Distance == x[5]
& carabid$Month %in% pair),12,replace=T),
                                   sample(which(carabid$Distance == x[6]
& carabid$Month %in% pair),12,replace=T),
                                   sample(which(carabid$Distance == x[7]
& carabid$Month %in% pair),12,replace=T))
      occ <- carabid[subs,variable] #permuted relative activity values
      grad <- carabid[subs,"Distance"] #transect positions of permuted
samples
      hof.rnd <- HOF(occ,grad,family = gaussian,boot= NULL,selectMethod =
"IC.weight", modeltypes=types,M=1,lim=100,test="Dev")  #HOF model
      hof.pred[,i] <-
predict(hof.rnd,model=pick.model(hof.rnd,test="Dev",modeltypes
=types),newdata = c(-20:20))   #store prediction from model to matrix
      }

      occ.obs.yr <- carabid[carabid$Month %in% pair,variable] #permuted
relative activity values
      grad.yr <- carabid[carabid$Month %in% pair,"Distance"] #transect
positions of permuted samples
      hof.yr <- HOF(occ.obs.yr,grad.yr,family = gaussian,boot=
NULL,selectMethod = "IC.weight", modeltypes=types,M=1,lim=100,test="Dev")
#HOF model
      hof.pred.yr <-
predict(hof.rnd,model=pick.model(hof.rnd,test="Dev",modeltypes
=types),newdata = c(-20:20))   #store prediction from model to matrix



      pair.2 <- month.sel[k]  #month selected for comparison, same as
previos
      hof.pred.2 <- matrix(data= NA, nrow = length(-20:20), ncol =
bootnr)
      for(i in 1:bootnr){subs.2 <- c(sample(which(carabid$Distance ==
x[1] & carabid$Month %in% pair.2),4,replace=T), #sample values with
replacement for each transect position
                                     sample(which(carabid$Distance ==
x[2] & carabid$Month %in% pair.2),4,replace=T),
                                     sample(which(carabid$Distance ==
x[3] & carabid$Month %in% pair.2),4,replace=T),
                                     sample(which(carabid$Distance ==
x[4] & carabid$Month %in% pair.2),4,replace=T),
```

```
                                     sample(which(carabid$Distance ==
x[5] & carabid$Month %in% pair.2),4,replace=T),
                                     sample(which(carabid$Distance ==
x[6] & carabid$Month %in% pair.2),4,replace=T),
                                     sample(which(carabid$Distance ==
x[7] & carabid$Month %in% pair.2),4,replace=T))
      occ.2 <- carabid[subs.2,variable]
      grad.2 <- carabid[subs.2,"Distance"]
      hof.rnd.2 <- HOF(occ.2,grad.2,family = gaussian,boot=
NULL,selectMethod = "IC.weight", modeltypes=types,M=1,lim=100,test="Dev")
      hof.pred.2[,i] <-
predict(hof.rnd.2,model=pick.model(hof.rnd.2,test="Dev",modeltypes
=types),newdata = c(-20:20))
      }

      #HOF for original data
      occ.obs <- carabid[which(carabid$Month == pair.2),variable]
      grad.obs <- carabid[which(carabid$Month == pair.2),"Distance"]
      hof.obs <- HOF(occ.obs,grad.obs,family = gaussian,boot=
NULL,selectMethod = "IC.weight", modeltypes=types,M=1,lim=100,test="Dev")
      hof.obs.pred <-
predict(hof.rnd.2,model=pick.model(hof.rnd.2,test="Dev",modeltypes
=types),newdata = c(-20:20))

      #plot results
      if(k == 1) par(mar=c(3,4.5,0.5,0.5)) # make space for labels in
first column
      if(k > 1) par(mar=c(3,3,0.5,0.5))
      if(k == 1 & j == 4) par(mar=c(4.5,4.5,0.5,0.5)) # make space for
labels in first column of last row
      if(k > 1 & j == 4) par(mar=c(4.5,3,0.5,0.5)) # make space for
labels in last row

      ylim <- max(carabid[,variable]) #set ylim for a row using maximum
observed value of variable
      plot(seq(-20,20,1),
apply(hof.pred,1,quantile,0.5),ylim=c(0,ylim),type="n",xlab=NA,ylab=NA,ma
in=NA,cex.axis=1.1)
      grid(lty=3,col="gray60")
      #text(-20,ylim*0.9,c(month.sel)[k],cex=1.2,pos=4) code by months
      text(-20,ylim*0.9,LETTERS[(j-1)*length(month.sel) +
k],cex=1.2,pos=4) #code by letters
      if(k == 1) mtext(var.names[j],2,3,cex=1.1) #y axis labels

      polygon(c(-20:20,20:-20),
c(apply(hof.pred,1,quantile,0.025),rev(apply(hof.pred,1,quantile,0.975)))
,col="#00000030",border=NA)
      lines(-20:20, apply(hof.pred,1,median),lwd=2,lty=2) #median of
bootstrapped model predictions
      #lines(-20:20, hof.pred.yr,lwd=2,lty=2) #observed model prediction

      polygon(c(-20:20,20:-20),
c(apply(hof.pred.2,1,quantile,0.025),rev(apply(hof.pred.2,1,quantile,0.97
5))),col="#AD111160",border=NA)
      lines(-20:20, apply(hof.pred.2,1,median),lwd=2,lty=1,col=2) #median
of bootstrapped model predictions
      #lines(-20:20,hof.obs.pred,lwd=2,lty=1,col=2) #original data fit
```

```
	if(k == 3 & j == length(variables)) mtext("Distance from arable field-woodlot boundary (m)",1,3,cex=1.2,adj=1)
	}
}
dev.off()
```
